# The metabolomic plasma profile of myeloma patients is considerably different from healthy subjects and reveals potential new therapeutic targets

**DOI:** 10.1371/journal.pone.0202045

**Published:** 2018-08-10

**Authors:** Normann Steiner, Udo Müller, Roman Hajek, Sabina Sevcikova, Bojana Borjan, Karin Jöhrer, Georg Göbel, Andreas Pircher, Eberhard Gunsilius

**Affiliations:** 1 Laboratory for Tumor Biology & Angiogenesis, Department of Internal Medicine V (Hematology and Medical Oncology), Medical University of Innsbruck, Innsbruck, Austria; 2 Biocrates Life Sciences AG, Innsbruck, Austria; 3 Faculty of Medicine, University of Ostrava, Ostrava, Czech Republic; 4 Department of Hematooncology, University Hospital Ostrava, Ostrava, Czech Republic; 5 Babak Myeloma Group, Department of Pathological Physiology, Faculty of Medicine, Masaryk University, Brno, Czech Republic; 6 Department of Clinical Hematology, University Hospital Brno, Brno, Czech Republic; 7 Tyrolean Cancer Research Institute, Innsbruck, Austria; 8 Salzburg Cancer Research Institute, Laboratory for Immunological and Molecular Cancer Research, Salzburg, Austria; 9 Department of Medical Statistics, Informatics and Health Economics, Medical University of Innsbruck, Innsbruck, Austria; University of South Alabama Mitchell Cancer Institute, UNITED STATES

## Abstract

**Introduction:**

Multiple myeloma (MM), a malignant plasma cell disorder, is still an incurable disease. Thus, the identification of novel therapeutic targets is of utmost importance. Here, we evaluated the peripheral blood-based metabolic profile of patients with MM.

**Material & methods:**

Peripheral blood plasma levels of 188 endogenous metabolites, including amino acids, biogenic amines, acylcarnitines, glycerophospholipids, sphingomyelins, and hexoses were determined in patients with plasma cell dyscrasias: monoclonal gammopathy of undetermined significance, a precursor stage of MM (MGUS, n = 15), newly diagnosed MM, (NDMM, n = 32), relapsed/refractory MM (RRMM, n = 19) and in 25 healthy controls by mass spectrometry.

**Results:**

Patients with NDMM, RRMM and MGUS have a substantially different metabolomic profile than healthy controls. The amount of eight plasma metabolites significantly differs between the NDMM and MGUS group: free carnitine, acetylcarnitine, glutamate, asymmetric dimethylarginine (ADMA) and four phosphatidylcholine (PC) species. In addition, the levels of octadecanoylcarnitine, ADMA and six PCs were significantly different between RRMM and MGUS patients. 13 different concentrations of metabolites were found between RRMM and NDMM patients (free carnitine, acetylcarnitine, creatinine, five LysoPCs and PCs). Pathway analyses revealed a distinct metabolic profile with significant alterations in amino acid, lipid, and energy metabolism in healthy volunteers compared to MGUS/MM patients.

**Conclusion:**

We identified different metabolic profiles in MGUS und MM patients in comparison to healthy controls. Thus, different metabolic processes, potentially the immunoregulation by indoleamine 2,3 dioxygenase-1 (IDO), which is involved in cancer development and progression supporting inflammatory processes in the tumor microenvironment and glutaminolysis, can serve as novel promising therapeutic targets in MM.

## Introduction

Multiple myeloma (MM) still remains an incurable malignancy of antibody-producing plasma cells. Monoclonal gammopathy of undetermined significance (MGUS) is a common asymptomatic precursor state of MM. Process of myeloma initiation is mediated by interaction of inherited genetic and environmental factors [1]. Clinical manifestations of MM occur due to proliferation of malignant plasma cells or effects of aberrant proteins released by myeloma cells. Most common signs and symptoms of MM include hypercalcemia, renal function impairment, anemia and lytic bone disease [2]. Major progress in treatment of multiple myeloma has been seen mainly due to development of novel agents, as proteasome inhibitors. Although survival of MM has increased, most of the patients ultimately relapse and become refractory [3]. Therefore, novel therapeutic options are needed. Dysregulated metabolism has been considered as one of the hallmarks of cancer [4]. The “Warburg effect” gave the first insights into the altered metabolism of cancer cells reporting that tumor cells prefer aerobic glycolysis for nutrient production and proliferation [5]. Recently, the field of metabolomics is attracting considerable interest due to novel methods and technical progress, which enable high quality measurements and contributed significantly to a better understanding of metabolic rewiring in pathological diseases as cancer [4]. Metabolic profiling in cancer emerges as a tool for the diagnosis, classification, treatment decisions and assessment of treatment efficacy, and identification of novel therapeutic targets. Metabolic profiling can be performed in different tissue samples and biofluids, such as serum, plasma, saliva, urine and in a high throughput fashion [6]. Nevertheless, metabolomics reporting and data analysis is not yet standardized and often more technical oriented and less biology driven [7].

The first metabolic analysis of MM cells identified that MM depend on glucose and glutamine metabolism [8]. Higher levels of isoleucine, arginine, acetate, phenylalanine, and tyrosine, and decreased levels of 3-hydroxybutyrate, lysine, glutamine, and some lipids were observed in myeloma patients at diagnosis, but not after achieving remission [9]. Bajpai et al. showed that targeting glutamine metabolism sensitizes MM cells to the bcl-2 inhibitor venetoclax. Cellular metabolic investigations revealed LDHA and HIF1α as novel targets for drug resistance in MM under bone marrow hypoxic conditions. Inhibition of LDHA and HIF1A can restore sensitivity to therapeutic drugs such as bortezomib [10]. Ludwig et al. highlights alterations in bone marrow metabolism as an early feature of the development of MGUS and MM [11]. Despite the interest in metabolomics of MM, the role and potential application in diagnostics, classification and prediction of therapy response remains unclear. Therefore, this study focuses to determine differences in the metabolic phenotype between healthy subjects, MGUS-, NDMM- and RRMM patients.

## Material and methods

### Ethics statement

Investigations have been conducted in accordance with the Declaration of Helsinki and the national and international guidelines and have been approved by the authors’ institutional review board (Ethics committee of the Medical University of Innsbruck, Austria, number: 1085/2017 and Ethics committee of the Faculty of Medicine, Masaryk University, Brno, Czech Republic, number: 20/1/2011). Patients provided written informed consent.

### Patients and sample collection

Patients with monoclonal gammopathy of undetermined significance (MGUS, n = 15), newly diagnosed MM (NDMM, n = 32) and relapsed/refractory MM (RRMM, n = 19) according to the International Myeloma Working Group (IMWG) criteria [12], were included in the study. All relevant clinical data and disease characteristics are shown in Table 1. Peripheral blood samples underwent centrifugation for 10 min at 1000-x *g* and obtained peripheral blood plasma was collected and stored at -80°C.

**Table 1:** Patient demographics and characteristics (n = 66).

Parameter	MGUS		NDMM		RRMM	
	n = 15	%	n = 32	%	n = 19	%
Median age (range), years	66 (52–72)		73 (60–80)		62 (55–68)	
Sex f/m						
F	4	27	15	47	10	53
M	11	73	17	53	9	47
ISS						
I			6	19	4	21
II			6	19	8	42
III			20	62	7	37
Type of Ig heavy chain (serum)						
IgG	13	87	16	50	11	58
IgM	0	0	0	0	2	10.5
IgA	2	13	7	22	2	10.5
IgD	0	0	1	3	0	0
Light chain only	0	0	8	25	4	21
Type of Ig light chain (serum)						
Kappa	8	53	19	59	10	53
Lambda	7	47	13	41	9	47
β-2 microglobulin >UNV	9	64	26	87	15	79
LDH >UNV	4	27	6	19	5	26
Creatinine ≥1.3 mg/dl	5	33	21	66	7	37
Serum calcium >UNV	0	0	6	19	3	16
Haemoglobin ≤12 g/dl	8	53	27	84	14	74
Platelets <100,000/mm^3^	1	7	7	22	12	63
Osteolytic bone lesions	1	7	27	84	19	100
Cytogenetic standard risk	4	27	17	53	5	26
Cytogenetic high risk	2	13	14	44	13	69
Cytogenetic not available	9	60	1	3	1	5
Therapy lines at sample collection						
1^st^ line	0	0	0	0	0	0
2^nd^ line	1	7	0	0	3	16
3^rd^ line	0	0	0	0	7	37
4^th^ line	0	0	0	0	2	11
5^th^ line	0	0	0	0	2	11
6^th^ line	0	0	0	0	4	21
7th line	0	0	0	0	1	5
1–3 therapy lines	1	7	0	0	10	53
≥ 4 therapy lines	0	0	0	0	9	47
BTZ based therapy	0	0	0	0	11	58
IMiD based therapy	1	7	0	0	7	37
Other therapies	0	0	0	0	1	5
No therapy	14	93	32	100	0	0

N, number of patients; ISS, International staging system; Ig, Immunoglobulin; UNV, upper normal value; LDH, lactate dehydrogenases; IMiD, Immunomodulatory drugs; BTZ, Bortezomib

### Metabolite analysis

A targeted metabolomics approach with the AbsoluteIDQ^™^ p180 kit (BIOCRATES Life Sciences AG, Innsbruck, Austria) was used for quantification, based on electrospray ionization liquid (ESI-LC-MS/MS) and flow-injection analysis mass spectrometry (FIA/MS) measurements. The assay allows simultaneous quantification of in total 188 metabolites out of 10 μL peripheral blood plasma, including amino acids, biogenic amines, acylcarnitines, glycerophospholipids, sphingomyelins, and the sum of hexoses. Analytic measurements were carried out on an API 4000 and API 5500 LC-MS/MS System (AB Sciex Deutschland GmbH, Darmstadt, Germany) controlled by the Analyst 1.5.1 and Biocrates Met*IDQ* software. For the calculation of metabolite concentrations, external standards served as a reference. Concentrations of all metabolites were calculated in μM and normalized with respect to internal quality control samples.

### Metabolomic data pre-processing and statistical analysis

All multivariate (Principal Component Analyses, PCA and Partial-Least-Squares Discrimination Analysis, PLS-DA and Hierarchical Cluster Analysis, HCA) and univariate statistical analyses were performed with the statistic platform R (Version 3.2.4). The raw data was cleaned applying a modified 80% rule (at least 80% valid values above the limit of detection (LOD) need to be available per analyte in the samples for each group), which resulted in exclusion of 48 metabolites (S1 Table). Remaining values below LOD in the dataset were replaced applying a logspline imputation method with values between LOD and LOD/2 (Parts A and B in S2 Table) [13]**.** To meet assumptions of statistical tests, data were additionally log2 transformed. Analysis of variance (ANOVA) and post-hoc t-tests were performed to identify significant metabolite alterations between the different patient groups and healthy controls. Data were corrected for multiple testing using the Benjamini–Hochberg (BH) procedure. Metabolites with p-values below a significance level of α = 0.05 were considered as statistically significant.

## Results

### 1. Supervised metabolic analysis of MM patients versus healthy controls

**Fig 1:**
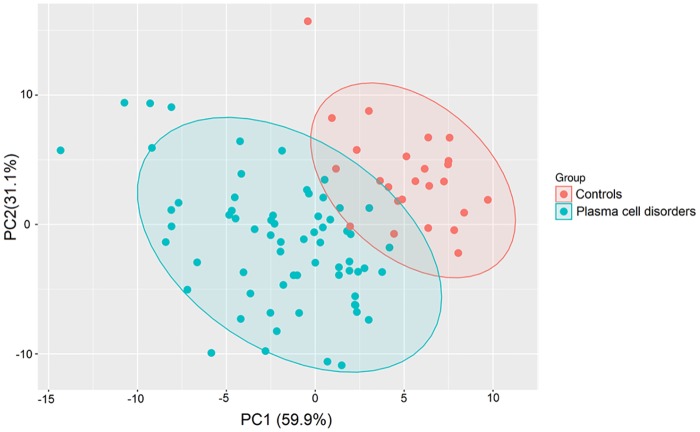
Multivariate PLS-DA of the metabolomic dataset. MM patients versus healthy controls. PLS-DA was applied on the cleaned, imputed and log2 transformed data set. 95% confidence interval ellipses are shown for the different groups.

Multivariate supervised PLS-DA, that relies on the class membership of each observation, was used on the transformed metabolomic data set (in total 188 metabolites) to identify overall differences. First PLS-DA was applied to compare peripheral blood plasma samples of healthy controls with those of the MM group (MGUS+NDMM+RRMM). As shown in the score-plot (Fig 1), the samples from controls were largely separated from the MM group. This finding indicates clearly a distinct metabolic composition of these two populations.

**Fig 2:**
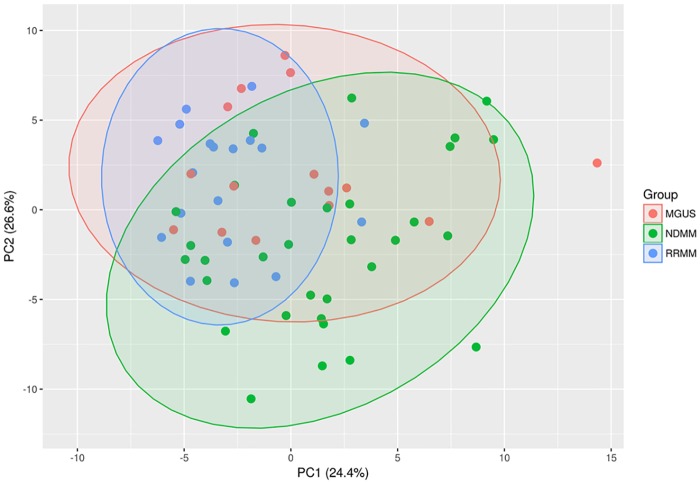
Multivariate PLS-DA of the metabolomic dataset. Separation between MGUS-, NDMM- and RRMM patients. PLS-DA was applied on the cleaned, imputed and log2 transformed data set. 95% confidence interval ellipses are shown for the different groups.

Second, PLS-DA was used to detect separations between MGUS-, NDMM- and RRMM patients (Fig 2). Because of the high complexity and in-group variation in the data, differences based on the largest principle components PC1 and PC2, were not as prominent as compared to controls. The substantial overlap remaining between the three patient groups indicates an overall similar metabolic composition. The difficulty in separating these three individual groups by the measured metabolites is likely due to the fact that their differences that segregate the samples are reduced by the intrinsic variations found from patient to patient.

In addition, based on the known percentage of plasma cells in the bone marrow of MM patients, we evaluated differences in the metabolic profile with respect to this parameter. In total, 18 metabolites from various classes such as the branched-chain amino acids (BCAAs; isoleucine, leucine and valine), various medium- and long-chain acylcarnitines (C10, C16 and C18) were significantly altered between the plasma cell ranges (S3 Table).

### 2. Metabolite alterations among the healthy controls and MGUS/MM patients

Student’s t-tests, fold change calculations and biochemical pathway mapping were performed in the following data analysis step to gain more precise insights about metabolic alterations between the four different groups.

**Fig 3:**
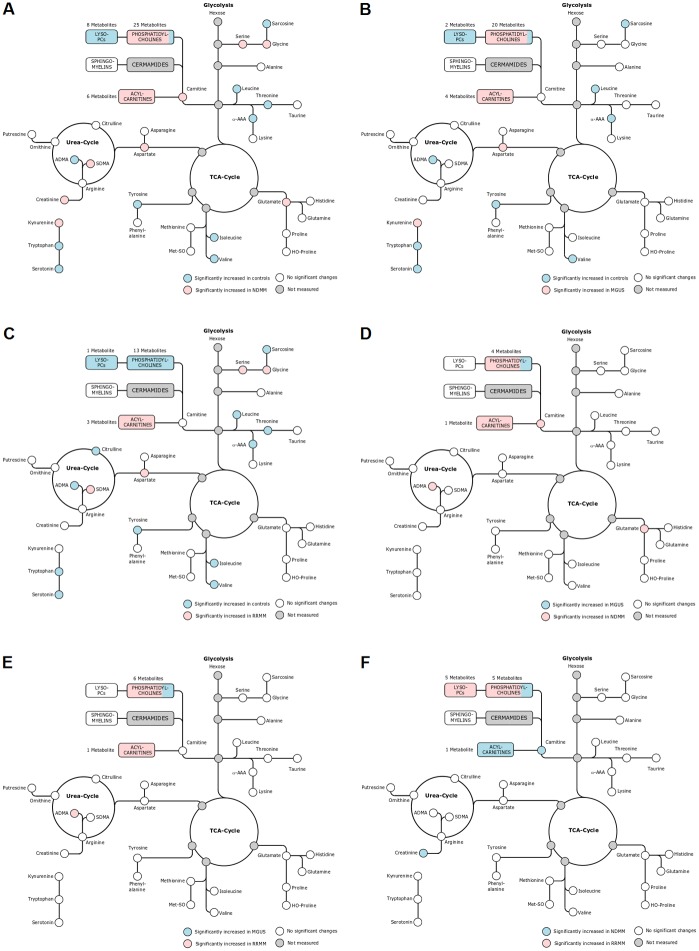
A) Pathways representation of significantly altered metabolites between healthy controls and NDMM. B) Pathways representation of significantly altered metabolites between healthy controls and MGUS. C) Pathways representation of significantly altered metabolites between healthy controls and RRMM. D) Pathways representation of significantly altered metabolites between MGUS and NDMM. E) Pathways representation of significantly altered metabolites between MGUS and RRMM. F) Pathways representation of significantly altered metabolites between NDMM and RRMM. Measured metabolites of the different pathways including glycolysis, TCA-Cycle and Urea Cycle are shown in circles. Statistically significant single metabolites or metabolites within a specific biochemical class (LysoPCs, PCs, Sphingomyelins, Acylcarnitines) are highlighted in blue and red.

57 metabolites were found to be significantly different between healthy controls and NDMM patients. These plasma metabolites comprised free carnitine (C0), acetylcarnitine (C2) as well as five long-chain acylcarnitines (C14:1, C16, C18, C18:1 and C18:2), which were all increased in the NDMM population (Fig 3A and Part A in S1 Fig). 36 metabolites from different biochemical pathways were significantly altered between controls and MGUS patients (Fig 3B and Part B in S1 Fig). Notably, the metabolic profile of the patient receiving an IMiD-based treatment did not differ significantly from other subjects in the MGUS group.

Prominently, three branched-chain amino acids (leucine, isoleucine, and valine) displayed low concentrations in NDMM patients, indicating a possible higher consumption of these metabolites (uptake by the MM cells in the bone marrow). In addition, glutamate, one of the most abundant amino acids in the human body, was significantly increased in NDMM patients compared to healthy controls, suggesting that MM cells in the bone marrow secrete or detoxify glutamate. In line, a recent meta-analysis of clinical metabolic profiling in blood and tissue of cancer patients identified glutamate as the most increased metabolite in blood as well as in tissue [7].

The activation of the kynurenine pathway occurs when the activity of indoleamine 2,3 dioxygenase-1 (IDO1), which catalyzes the conversion of tryptophan to kynurenine, is increased [14, 15]. This process reduces blood tryptophan levels, which in turn decrease the availability of a higher kynurenine/tryptophan ratio, as detected in the NDMM patients. In addition, eight lysophosphatidylcholines (LysoPCs) were significantly lower in NDMM patients. Out of the 25 significantly altered phosphatidylcholines (PCs), 23 were increased in NDMM samples. Metabolite differences in acylcarnitines, amino acids and biogenic amines between healthy controls and RRMM patients were comparable to those observed between controls and NDMM patients. 32 metabolites were significantly altered and long-chain acylcarnitines (C16, C18, C18:1) were elevated in RRMM patients in comparison to the control group (Fig 3C and Part C in S1 Fig). All three BCAAs and ADMA were increased in healthy subjects. The kynurenine/tryptophan ratio, reflecting possible increased IDO activity, was higher in RRMM patients.

Metabolic alterations between healthy controls and MGUS patients (in total 36 significant metabolites) were similar to the changes in the RRMM and NDMM groups, with significantly enriched plasma acylcarnitines in MGUS samples (C2, C18, C18:1 and C18:2). Increased kynurenine pathway activation and lower ADMA levels could be again detected in MGUS patients.

Differences between NDMM-, RRMM- and MGUS patients were not as prominent as compared to the healthy control group with fewer significantly affected metabolites. In total, eight metabolites were significantly altered between the NDMM and the MGUS group, including free carnitine, acetylcarnitine, glutamate, ADMA, and four PCs species (Fig 3D and Part D in S1 Fig). Eight metabolites (ADMA, octadecanoylcarnitine and 6 PCs) were significantly altered between RRMM- and MGUS patients (Fig 3E and Part E in S1 Fig). In RRMM- and NDMM patients the 13 significantly altered metabolites comprised free carnitine, acetylcarnitine, creatinine, several LysoPCs and PCs (Fig 3F and Part F in S1 Fig). Mean concentration and standard deviation (SD) of significantly altered metabolites are shown in Parts A-F in S4 Table.

## Discussion

MM is a heterogenic disease with a dynamic metabolic and proteomic process in bone marrow and its microenvironment [16]. In the literature only marginally reports exist about a specific metabolic profile in MM [10]. In this study we determined differences in metabolic phenotypes between healthy subjects, MGUS-, NDMM- and RRMM patients.

Using multivariate PLS-DA of peripheral blood plasma samples, we could clearly separate healthy controls from MGUS-, NDMM-, and RRMM patients. Thus, it appears that MM patients have a different metabolic profile than healthy subjects. We found 57 metabolites to be significantly different between healthy controls and NDMM patients. Free carnitine (C0), acetylcarnitine (C2) and five long-chain acylcarnitines (C14:1, C16, C18, C18:1 and C18:2) were all increased in the NDMM population. Carnitine is essential for the transport of long-chain acyl groups from fatty acids into the mitochondrial matrix. Inside the mitochondrial matrix fatty acids can be broken down to acetyl-CoA through a process called ß-oxidation, so they can enter the tricarboxylic acid (TCA) cycle for energy production [17]. Thus, acylcarnitines represent the carrier form of activated fatty acids for the transport across the inner mitochondrial membrane and alterations potentially point toward changes in lipid breakdown.

Moreover, 36 amino acids and biogenic amines were significantly increased or decreased between controls and MGUS patients. The three BCAAs (leucine, isoleucine, valine) had a lower concentration in NDMM patients. Notably, BCAAs are not only an essential nutrient source, but can also function as potent signaling molecules and changes in their levels have been linked to cancer progression [18]. BCAAs can be efficiently used for protein synthesis or oxidized for energy purposes by cancer cells. As BCAAs are essential, cancer fully rely on dietary BCAA intake or their release from protein degradation [19]. In addition, it has been shown that the enzymes catalyzing the first step in BCAA degradation are overexpressed in many cancer types [20, 21]. The cytosolic branched chain-amino transferase 1 (BCAT1), which converts BCAAs to their corresponding branched-chain a-keto acids is involved in cancer proliferation and has been proposed as a prognostic cancer marker [18, 22–24]. Moreover, a study investigating the metabolic profile of bortezomib resistance in cell culture and primary cells showed that the serine synthesis pathway (SSP) has significantly increased activity in bortezomib resistant MM and plasmacytoid lymphoma cell lines. Importantly, the study also observed a strong correlation between SSP activity and the ability of cells to withstand increasing bortezomib concentrations in all bortezomib resistant cell lines tested [25]. In this concern, it may be that serine and glycine were significantly increased in our NDMM- and RRMM patients.

Furthermore, kynurenine, which is converted from the essential aromatic amino acid tryptophan by the enzyme IDO1 plays an important role in several cellular functions. The activity of IDO and the activation of the kynurenine pathway, which is indirectly reflected by the kynurenine to tryptophan ratio, was elevated in NDMM patients. The IDO enzyme has been reported to be a key player in both cancer development and progression as it supports inflammatory processes in the tumor microenvironment, mediates immune tolerance to tumor antigens and suppresses T-cells and natural killer cells [26, 27]. In addition, the extent of the abnormality in tryptophan metabolism has been shown to directly correlate with the aggressiveness of the malignancy [28, 29]. Therefore, the results of this study corroborate that the aberrant kynurenine pathway activation by IDO may serve as a potential cellular mechanism, which is present in NDMM patients to induce a systemic deregulation of immune responses [30, 31]. Based on the evidence for immune tolerance, there has been increasing interest in IDO as a novel therapeutic target for the development of new anti-cancer and anti-myeloma drugs. Thus, promising IDO-inhibiting drugs for use in multiple myeloma are now the focus of research.

Glutamate, which was elevated in NDMM patients, is a nonessential amino acid and serves as the major bioenergetic substrate for proliferating normal but also neoplastic cells. Glutamate functions as an excitatory neurotransmitter that plays essential roles in metabolic, and oncogenic signaling pathways [32]. As an intermediate in glutaminolysis, glutamine is converted into TCA cycle metabolites through the activity of multiple enzymes. Aberrant glutaminolysis has been described as a hallmark of cancer, as many cancer cells undergo metabolic reprogramming that makes them highly glutamine dependent for their survival and proliferation [33, 34]. Especially, targeting the glutaminolysis pathway by inhibition of glutamine synthase (GS), cellular glutamine transporter (GLUTs) or oncogenes involved in the regulation of this metabolic pathway is a promising approach for heavily pretreated multiple myeloma patients [35].

Asymmetric dimethylarginine (ADMA), which was enriched in healthy controls, is an endogenous inhibitor of nitric oxide synthase (NOS) and is derived from methylation of arginine residues in proteins. Increased blood levels of ADMA have been linked to cardiovascular diseases, renal failure and hypertension, in which ADMA was described as an independent risk factor [36, 37].

Moreover, in our study we observed that eight lysophosphatidylcholines (LysoPCs) were significantly lower in NDMM patients. LysoPCs result from the partial hydrolysis of PCs, at which one fatty acid group is enzymatically removed by Phospholipase A2 [38]. LysoPCs are emerging as a novel class of inflammatory lipids, joining thromboxanes, leukotrienes and prostaglandins, with which they share common metabolic pathways and regulatory mechanisms.

Additionally, we found that 32 metabolites were significantly changed and again several long-chain acylcarnitines (C16, C18, C18:1) were elevated in the RRMM group, indicating again changes in overall lipid energy metabolism. In line with the NDMM group, all three BCAAs as well as ADMA were increased in the control samples, whereas a higher kynurenine/tryptophan ratio was present in RRMM patients, pointing towards an elevated kynurenine pathway activation. Compared to the NDMM group, a smaller number lysoPCs and PCs were significantly altered between controls and RRMM patients.

The metabolic alterations between healthy controls and MGUS patients were again similar to the changes in the RRMM- and NDMM groups, with significantly enriched peripheral blood plasma acylcarnitines in MGUS samples (C2, C18, C18:1 and C18:2). An increased kynurenine/tryptophan ratio and lower ADMA levels again could be detected in MGUS patients. Metabolic alterations of several different biochemical classes in the bone marrow environment from MGUS patients were recently reported [11]. These changes, especially for the lipid metabolism, could also be observed in our study with peripheral blood.

In our study we found significantly different changed metabolites in MGUS, NDMM and RRMM patients. Moreover, we identified a distinct metabolic profile with significant alterations in amino acid, lipid and energy metabolism in healthy volunteers compared to MGUS/MM patients. We assume that several cellular metabolic processes, most likely immunoregulation by IDO or glutaminolysis may serve as novel promising therapeutic targets in MM. Promising future treatments may combine approaches to target different metabolic pathways such as glutamine metabolism, glycolysis or the TCA cycle. Further investigations are needed to examine the association of certain metabolites with disease progression and drug resistance.

## Supporting information

S1 Table Metabolite data cleaning. 48 metabolites were removed from the dataset after application of an 80% rule.
(DOCX)

S2 Table A) Coefficients of variance based on quality control samples for the targeted metabolomics assay. B) Limit of detection (LOD) for each analyte of the targeted metabolomics assay.
(DOCX)

S3 Table Metabolite alterations in peripheral blood plasma compared to diverse bone marrow plasma cell ranges in MM patients. (DOCX)

S4 Table A) Mean concentration and standard deviation (SD) of metabolites significantly altered between healthy controls and MGUS. B) Mean concentration and standard deviation (SD) of metabolites significantly altered between healthy controls and NDMM. C) Mean concentration and standard deviation (SD) of metabolites significantly altered between healthy controls and RRMM. D) Mean concentration and standard deviation (SD) of metabolites significantly altered between NDMM and MGUS. E) Mean concentration and standard deviation (SD) of metabolites significantly altered between RRMM and MGUS. F) Mean concentration and standard deviation (SD) of metabolites significantly altered between RRMM and NDMM.
(DOCX)

S1 Fig A) heat maps of metabolites between healthy controls and NDMM. B) heat maps of metabolites between healthy controls and MGUS. C) heat maps of metabolites between healthy controls and RRMM. D) heat maps of metabolites between MGUS and NDMM. E) heat maps of metabolites between MGUS and RRMM. F) heat maps of metabolites between NDMM and RRMM.
(TIF)
