# Supplementary material for: The metabolomic plasma profile of myeloma patients is considerably different from healthy subjects and reveals potential new therapeutic targets
Source: PLoS One. 2018 Aug 10;13(8):e0202045. doi: 10.1371/journal.pone.0202045 (PMC6086450; doi:10.1371/journal.pone.0202045)
Supplement: S1 Table — 48 metabolites were removed from the dataset after application of an 80% rule. (DOCX) [file pone.0202045.s001.docx]

| **Biogenic Amines** | **Acylcarnitines** | | **Lipids** |
| --- | --- | --- | --- |
| Ac-Orn | C3-DC (C4-OH) | C10:1 | lysoPC a C14:0 |
| c4-OH-Pro | C3-OH | C10:2 | lysoPC a C24:0 |
| Carnosine | C3:1 | C12 | PC aa C26:0 |
| DOPA | C4:1 | C12-DC | PC aa C30:2 |
| Dopamine | C5 | C12:1 | PC aa C40:1 |
| Histamine | C5-DC (C6-OH) | C14 | PC ae C30:2 |
| Nitro-Tyr | C5-M-DC | C14:1-OH | PC ae C42:0 |
| PEA | C5-OH (C3-DC-M) | C14:2 | SM C22:3 |
| Spermidine | C5:1 | C14:2-OH |  |
| Spermine | C5:1-DC | C16-OH |  |
|  | C6 (C4:1-DC) | C16:1 |  |
|  | C6:1 | C16:1-OH |  |
|  | C7-DC | C16:2 |  |
|  | C8 | C16:2-OH |  |
|  | C9 | C18:1-OH |  |
|  | C10 |  |  |

**S1 Table**
