# Supplementary material for: The metabolomic plasma profile of myeloma patients is considerably different from healthy subjects and reveals potential new therapeutic targets
Source: PLoS One. 2018 Aug 10;13(8):e0202045. doi: 10.1371/journal.pone.0202045 (PMC6086450; doi:10.1371/journal.pone.0202045)
Supplement: S2 Table — A) Coefficients of variance based on quality control samples for the targeted metabolomics assay. B) Limit of detection (LOD) for each analyte of the targeted metabolomics assay. (DOCX) [file pone.0202045.s002.docx]

**S2 Table**

**A)**

| **Amino Acids and Biogenic Amines** | | **Acylcarnitines** | | **(Lyso-)phosphatidylcholines** | | | | **Sphingomyelins** | |
| --- | --- | --- | --- | --- | --- | --- | --- | --- | --- |
| **Metabolite** | **CV Quality Controls** | **Metabolite** | **CV Quality Controls** | **Metabolite** | **CV Quality Controls** | **Metabolite** | **CV Quality Controls** | **Metabolite** | **CV Quality Controls** |
| Ala | 4,79% | C0 | 5,14% | lysoPC a C14:0 | 2,24% | PC aa C40:6 | 4,10% | SM (OH) C14:1 | 6,67% |
| Arg | 4,99% | C2 | 5,23% | lysoPC a C16:0 | 5,69% | PC aa C42:0 | 5,06% | SM (OH) C16:1 | 8,22% |
| Asn | 5,49% | C3 | 6,55% | lysoPC a C16:1 | 2,68% | PC aa C42:1 | 5,56% | SM (OH) C22:1 | 4,74% |
| Asp | 6,55% | C3-DC (C4-OH) | 12,70% | lysoPC a C17:0 | 3,18% | PC aa C42:2 | 9,64% | SM (OH) C22:2 | 5,07% |
| Cit | 1,01% | C3-OH | 10,13% | lysoPC a C18:0 | 4,41% | PC aa C42:4 | 2,64% | SM (OH) C24:1 | 6,65% |
| Gln | 5,09% | C3:1 | 7,27% | lysoPC a C18:1 | 5,74% | PC aa C42:5 | 2,51% | SM C16:0 | 6,73% |
| Glu | 5,48% | C4 | 8,36% | lysoPC a C18:2 | 4,86% | PC aa C42:6 | 5,07% | SM C16:1 | 7,26% |
| Gly | 5,89% | C4:1 | 5,77% | lysoPC a C20:3 | 13,65% | PC ae C30:0 | 3,00% | SM C18:0 | 6,93% |
| His | 2,47% | C5 | 7,61% | lysoPC a C20:4 | 7,34% | PC ae C30:1 | 95,11% | SM C18:1 | 7,52% |
| Ile | 5,02% | C5-DC (C6-OH) | 10,71% | lysoPC a C24:0 | 10,06% | PC ae C30:2 | 21,15% | SM C20:2 | 10,01% |
| Leu | 3,37% | C5-M-DC | 19,43% | lysoPC a C26:0 | 79,43% | PC ae C32:1 | 6,64% | SM C22:3 | 84,76% |
| Lys | 4,43% | C5-OH (C3-DC-M) | 13,34% | lysoPC a C26:1 | 47,46% | PC ae C32:2 | 10,82% | SM C24:0 | 6,00% |
| Met | 4,32% | C5:1 | 4,84% | lysoPC a C28:0 | 52,95% | PC ae C34:0 | 7,29% | SM C24:1 | 4,61% |
| Orn | 7,39% | C5:1-DC | 3,00% | lysoPC a C28:1 | 67,81% | PC ae C34:1 | 4,71% | SM C26:0 | 9,88% |
| Phe | 5,81% | C6 (C4:1-DC) | 5,22% | PC aa C24:0 | 4,89% | PC ae C34:2 | 4,76% | SM C26:1 | 7,39% |
| Pro | 5,11% | C6:1 | 15,38% | PC aa C26:0 | 58,36% | PC ae C34:3 | 4,55% |  |  |
| Ser | 5,70% | C7-DC | 11,92% | PC aa C28:1 | 10,60% | PC ae C36:0 | 3,34% |  |  |
| Thr | 2,82% | C8 | 2,55% | PC aa C30:0 | 6,26% | PC ae C36:1 | 5,47% | **Hexoses** | **CV Quality Controls** |
| Trp | 6,45% | C9 | 12,64% | PC aa C30:2 | 6,49% | PC ae C36:2 | 4,31% | H1 | 5,28% |
| Tyr | 3,24% | C10 | 5,80% | PC aa C32:0 | 6,05% | PC ae C36:3 | 3,48% |  |  |
| Val | 5,11% | C10:1 | 6,28% | PC aa C32:1 | 4,76% | PC ae C36:4 | 5,46% |  |  |
| Ac-Orn | 5,78% | C10:2 | 3,40% | PC aa C32:2 | 6,30% | PC ae C36:5 | 4,47% |  |  |
| ADMA | 3,17% | C12 | 6,81% | PC aa C32:3 | 11,02% | PC ae C38:0 | 2,80% |  |  |
| alpha-AAA | 1,03% | C12-DC | 4,93% | PC aa C34:1 | 4,78% | PC ae C38:1 | 18,39% |  |  |
| c4-OH-Pro | 5,27% | C12:1 | 6,73% | PC aa C34:2 | 4,79% | PC ae C38:2 | 5,18% |  |  |
| Carnosine | 1,86% | C14 | 5,03% | PC aa C34:3 | 4,40% | PC ae C38:3 | 3,06% |  |  |
| Creatinine | 3,91% | C14:1 | 8,59% | PC aa C34:4 | 4,50% | PC ae C38:4 | 4,96% |  |  |
| DOPA | 2,90% | C14:1-OH | 5,82% | PC aa C36:0 | 6,01% | PC ae C38:5 | 4,72% |  |  |
| Dopamine | 1,68% | C14:2 | 14,33% | PC aa C36:1 | 4,58% | PC ae C38:6 | 3,29% |  |  |
| Histamine | 4,08% | C14:2-OH | 12,33% | PC aa C36:2 | 4,81% | PC ae C40:1 | 7,52% |  |  |
| Kynurenine | 4,29% | C16 | 5,83% | PC aa C36:3 | 4,43% | PC ae C40:2 | 3,99% |  |  |
| Met-SO | 4,50% | C16-OH | 13,74% | PC aa C36:4 | 4,31% | PC ae C40:3 | 2,69% |  |  |
| Nitro-Tyr | 5,26% | C16:1 | 4,22% | PC aa C36:5 | 3,98% | PC ae C40:4 | 5,10% |  |  |
| PEA | 4,55% | C16:1-OH | 12,88% | PC aa C36:6 | 5,82% | PC ae C40:5 | 4,01% |  |  |
| Putrescine | 10,13% | C16:2 | 14,43% | PC aa C38:0 | 3,46% | PC ae C40:6 | 4,99% |  |  |
| Sarcosine | 2,30% | C16:2-OH | 22,69% | PC aa C38:1 | 17,09% | PC ae C42:0 | 7,12% |  |  |
| SDMA | 3,47% | C18 | 5,40% | PC aa C38:3 | 4,16% | PC ae C42:1 | 17,80% |  |  |
| Serotonin | 7,21% | C18:1 | 1,49% | PC aa C38:4 | 4,64% | PC ae C42:2 | 6,84% |  |  |
| Spermidine | 3,63% | C18:1-OH | 20,54% | PC aa C38:5 | 4,68% | PC ae C42:3 | 6,92% |  |  |
| Spermine | 6,93% | C18:2 | 4,64% | PC aa C38:6 | 3,97% | PC ae C42:4 | 7,09% |  |  |
| t4-OH-Pro | 3,65% |  |  | PC aa C40:1 | 8,09% | PC ae C42:5 | 3,05% |  |  |
| Taurine | 5,36% |  |  | PC aa C40:2 | 5,97% | PC ae C44:3 | 12,52% |  |  |
|  |  |  |  | PC aa C40:3 | 4,32% | PC ae C44:4 | 7,22% |  |  |
|  |  |  |  | PC aa C40:4 | 4,92% | PC ae C44:5 | 1,18% |  |  |
|  |  |  |  | PC aa C40:5 | 3,74% | PC ae C44:6 | 3,31% |  |  |

**B)**

| **Amino Acids and Biogenic Amines** | | **Acylcarnitines** | | **Lyso(phosphtaidylcholines)** | | | | **Sphingomyelins** | |
| --- | --- | --- | --- | --- | --- | --- | --- | --- | --- |
| Ala | 1 | C0 | 1,98 | lysoPC a C14:0 | 12,9 | PC aa C40:6 | 0,2867 | SM (OH) C14:1 | 0,025 |
| Arg | 0,5 | C2 | 0,3278 | lysoPC a C16:0 | 0,0592 | PC aa C42:0 | 0,0354 | SM (OH) C16:1 | 0,012 |
| Asn | 1,5 | C3 | 0,1122 | lysoPC a C16:1 | 0,0966 | PC aa C42:1 | 0,0073 | SM (OH) C22:1 | 0,0025 |
| Asp | 1,5 | C3-DC (C4-OH) | 0,1489 | lysoPC a C17:0 | 0,0254 | PC aa C42:2 | 0,0753 | SM (OH) C22:2 | 0,01 |
| Cit | 1 | C3-OH | 0,0874 | lysoPC a C18:0 | 0,2205 | PC aa C42:4 | 0,0069 | SM (OH) C24:1 | 0,0019 |
| Gln | 1,5 | C3:1 | 0,0459 | lysoPC a C18:1 | 0,054 | PC aa C42:5 | 0,0032 | SM C16:0 | 0,0087 |
| Glu | 2 | C4 | 0,0926 | lysoPC a C18:2 | 0,0305 | PC aa C42:6 | 0,1208 | SM C16:1 | 0,005 |
| Gly | 0,5 | C4:1 | 0,077 | lysoPC a C20:3 | 0,0894 | PC ae C30:0 | 0,0961 | SM C18:0 | 0,0177 |
| His | 0,5 | C5 | 0,1098 | lysoPC a C20:4 | 0,0125 | PC ae C30:1 | 0,02 | SM C18:1 | 0,006 |
| Ile | 13,2 | C5-DC (C6-OH) | 0,0595 | lysoPC a C24:0 | 0,7472 | PC ae C30:2 | 0,291 | SM C20:2 | 0,0002 |
| Leu | 93,6 | C5-M-DC | 0,088 | lysoPC a C26:0 | 0,0152 | PC ae C32:1 | 0,001 | SM C22:3 | 0,01 |
| Lys | 0,5 | C5-OH (C3-DC-M) | 0,1052 | lysoPC a C26:1 | 0,0432 | PC ae C32:2 | 0,0253 | SM C24:0 | 0,0038 |
| Met | 0,1 | C5:1 | 0,0919 | lysoPC a C28:0 | 0,0938 | PC ae C34:0 | 0,0067 | SM C24:1 | 0,0017 |
| Orn | 0,5 | C5:1-DC | 0,0858 | lysoPC a C28:1 | 0,0115 | PC ae C34:1 | 0,008 | SM C26:0 | 0,0026 |
| Phe | 0,1 | C6 (C4:1-DC) | 0,076 | PC aa C24:0 | 0,0262 | PC ae C34:2 | 0,0027 | SM C26:1 | 0,006 |
| Pro | 1 | C6:1 | 0,0605 | PC aa C26:0 | 0,9002 | PC ae C34:3 | 0,0113 |  |  |
| Ser | 1 | C7-DC | 0,0503 | PC aa C28:1 | 0,0295 | PC ae C36:0 | 0,1214 | **Hexoses** | |
| Thr | 0,5 | C8 | 0,1834 | PC aa C30:0 | 0,1249 | PC ae C36:1 | 0,0504 | H1 | 20,4 |
| Trp | 0,5 | C9 | 0,0372 | PC aa C30:2 | 0,006 | PC ae C36:2 | 0,0246 |  |  |
| Tyr | 0,5 | C10 | 0,1052 | PC aa C32:0 | 0,0085 | PC ae C36:3 | 0,0024 |  |  |
| Val | 0,5 | C10:1 | 0,2945 | PC aa C32:1 | 0,002 | PC ae C36:4 | 0,0189 |  |  |
| Ac-Orn | 0,2 | C10:2 | 0,1784 | PC aa C32:2 | 0,0054 | PC ae C36:5 | 0,005 |  |  |
| ADMA | 0,08 | C12 | 0,137 | PC aa C32:3 | 0,0018 | PC ae C38:0 | 0,0949 |  |  |
| alpha-AAA | 0,4 | C12-DC | 0,2703 | PC aa C34:1 | 0,0081 | PC ae C38:1 | 0,0034 |  |  |
| c4-OH-Pro | 0,0285 | C12:1 | 0,1555 | PC aa C34:2 | 0,007 | PC ae C38:2 | 0,0025 |  |  |
| Carnosine | 0,1 | C14 | 0,0376 | PC aa C34:3 | 0,0053 | PC ae C38:3 | 0,006 |  |  |
| Creatinine | 1 | C14:1 | 0,0127 | PC aa C34:4 | 0,0055 | PC ae C38:4 | 0,01 |  |  |
| DOPA | 0,2 | C14:1-OH | 0,0219 | PC aa C36:0 | 0,3057 | PC ae C38:5 | 0,0037 |  |  |
| Dopamine | 0,1 | C14:2 | 0,0209 | PC aa C36:1 | 0,0055 | PC ae C38:6 | 0,0059 |  |  |
| Histamine | 0,01 | C14:2-OH | 0,0181 | PC aa C36:2 | 0,0116 | PC ae C40:1 | 0,0109 |  |  |
| Kynurenine | 0,693 | C16 | 0,0124 | PC aa C36:3 | 0,0047 | PC ae C40:2 | 0,0012 |  |  |
| Met-SO | 0,3 | C16-OH | 0,0143 | PC aa C36:4 | 0,0032 | PC ae C40:3 | 0,006 |  |  |
| Nitro-Tyr | 3,45 | C16:1 | 0,2957 | PC aa C36:5 | 0,0055 | PC ae C40:4 | 0,0554 |  |  |
| PEA | 0,02 | C16:1-OH | 0,0189 | PC aa C36:6 | 0,0002 | PC ae C40:5 | 0,0008 |  |  |
| Putrescine | 0,02 | C16:2 | 0,0142 | PC aa C38:0 | 0,0243 | PC ae C40:6 | 0,0041 |  |  |
| Sarcosine | 0,3 | C16:2-OH | 0,0268 | PC aa C38:1 | 0,0045 | PC ae C42:0 | 0,7004 |  |  |
| SDMA | 0,03 | C18 | 0,0129 | PC aa C38:3 | 0,0028 | PC ae C42:1 | 0,082 |  |  |
| Serotonin | 0,03 | C18:1 | 0,0447 | PC aa C38:4 | 0,0056 | PC ae C42:2 | 0,0081 |  |  |
| Spermidine | 0,2628 | C18:1-OH | 0,0346 | PC aa C38:5 | 0,0056 | PC ae C42:3 | 0,0037 |  |  |
| Spermine | 0,08 | C18:2 | 0,0112 | PC aa C38:6 | 0,02 | PC ae C42:4 | 0,3 |  |  |
| t4-OH-Pro | 0,1 |  |  | PC aa C40:1 | 0,3609 | PC ae C42:5 | 1,1 |  |  |
| Taurine | 0,8 |  |  | PC aa C40:2 | 0,0083 | PC ae C44:3 | 0,0279 |  |  |
|  |  |  |  | PC aa C40:3 | 0,0041 | PC ae C44:4 | 0,0846 |  |  |
|  |  |  |  | PC aa C40:4 | 0,0075 | PC ae C44:5 | 0,0654 |  |  |
|  |  |  |  | PC aa C40:5 | 0,04 | PC ae C44:6 | 0,0203 |  |  |
