# Supplementary material for: The metabolomic plasma profile of myeloma patients is considerably different from healthy subjects and reveals potential new therapeutic targets
Source: PLoS One. 2018 Aug 10;13(8):e0202045. doi: 10.1371/journal.pone.0202045 (PMC6086450; doi:10.1371/journal.pone.0202045)
Supplement: S3 Table — (DOCX) [file pone.0202045.s003.docx]

**S3 Table**
