# Supplementary material for: The metabolomic plasma profile of myeloma patients is considerably different from healthy subjects and reveals potential new therapeutic targets
Source: PLoS One. 2018 Aug 10;13(8):e0202045. doi: 10.1371/journal.pone.0202045 (PMC6086450; doi:10.1371/journal.pone.0202045)
Supplement: S4 Table — A) Mean concentration and standard deviation (SD) of metabolites significantly altered between healthy controls and MGUS. B) Mean concentration and standard deviation (SD) of metabolites significantly altered between healthy controls and NDMM. C) Mean concentration and standard deviation (SD) of metabolites significantly altered between healthy controls and RRMM. D) Mean concentration and standard deviation (SD) of metabolites significantly altered between NDMM and MGUS. E) Mean concentration and standard deviation (SD) of metabolites significantly altered between RRMM and MGUS. F) Mean concentration and standard deviation (SD) of metabolites significantly altered between RRMM and NDMM. (DOCX) [file pone.0202045.s004.docx]

| **Metabolite** | **Mean Conc. Control [µM]** | **SD Controls** | **Mean Conc. MGUS [µM]** | **SD MGUS** | **p-value: MGUS-Control** | **Fold Change: MGUS-Control** |
| --- | --- | --- | --- | --- | --- | --- |
| Sarcosine | 5,32 | 2,845 | 1,44 | 0,785 | 6,18E-08 | 0,27 |
| PC.aa.C36.1 | 37,18 | 5,3 | 23,00 | 6,751 | 0,0001 | 0,62 |
| lysoPC.a.C26.1 | 0,12 | 0,016 | 0,06 | 0,029 | 0,0001 | 0,50 |
| Serotonin | 0,27 | 0,141 | 0,08 | 0,094 | 0,0003 | 0,30 |
| alpha.AAA | 1,68 | 0,89 | 0,65 | 0,574 | 0,0003 | 0,39 |
| PC.aa.C38.3 | 37,94 | 4,674 | 23,92 | 6,911 | 0,0003 | 0,63 |
| Tyr | 103,99 | 32,14 | 70,69 | 19,94 | 0,0009 | 0,68 |
| PC.aa.C34.1 | 146,42 | 13,868 | 106,06 | 25,772 | 0,0010 | 0,72 |
| Leu | 202,25 | 34,655 | 147,40 | 29,034 | 0,0014 | 0,73 |
| PC.aa.C36.3 | 91,36 | 9,282 | 63,20 | 18,684 | 0,0016 | 0,69 |
| Trp | 74,03 | 15,756 | 52,48 | 14,105 | 0,0020 | 0,71 |
| Asp | 2,48 | 2,013 | 6,63 | 3,986 | 0,0021 | 2,67 |
| PC.aa.C36.2 | 147,17 | 49,539 | 104,35 | 31,773 | 0,0024 | 0,71 |
| PC.aa.C40.5 | 8,24 | 1,336 | 5,66 | 1,69 | 0,0027 | 0,69 |
| PC.aa.C32.1 | 13,31 | 2,701 | 7,68 | 3,128 | 0,0027 | 0,58 |
| PC.aa.C36.5 | 28,90 | 7,891 | 15,02 | 12,223 | 0,0028 | 0,52 |
| Val | 339,60 | 40,583 | 265,40 | 49,647 | 0,0043 | 0,78 |
| PC.aa.C34.3 | 12,03 | 1,438 | 7,58 | 3,593 | 0,0044 | 0,63 |
| PC.aa.C36.6 | 0,90 | 0,317 | 0,49 | 0,261 | 0,0065 | 0,54 |
| C2 | 5,20 | 1,722 | 6,69 | 1,451 | 0,0071 | 1,29 |
| C18.2 | 0,03 | 0,007 | 0,05 | 0,021 | 0,0090 | 1,67 |
| PC.aa.C40.6 | 22,57 | 4,75 | 14,97 | 5,796 | 0,0098 | 0,66 |
| PC.aa.C38.5 | 43,90 | 5,757 | 30,95 | 10,328 | 0,0112 | 0,71 |
| C18.1 | 0,09 | 0,003 | 0,14 | 0,056 | 0,0119 | 1,56 |
| PC.aa.C24.0 | 0,06 | 0,033 | 0,09 | 0,03 | 0,0147 | 1,50 |
| Kynurenine | 2,24 | 0,964 | 4,26 | 2,754 | 0,0176 | 1,90 |
| PC.aa.C32.3 | 0,34 | 0,083 | 0,25 | 0,087 | 0,0215 | 0,74 |
| lysoPC.a.C20.3 | 1,76 | 0,367 | 1,23 | 0,527 | 0,0230 | 0,70 |
| ADMA | 0,36 | 0,121 | 0,47 | 0,161 | 0,0234 | 1,31 |
| C18 | 0,03 | 0,007 | 0,04 | 0,016 | 0,0247 | 1,33 |
| PC.aa.C34.4 | 1,35 | 0,4 | 0,84 | 0,44 | 0,0279 | 0,62 |
| PC.ae.C38.0 | 1,77 | 0,083 | 1,23 | 0,445 | 0,0322 | 0,69 |
| PC.aa.C32.2 | 2,53 | 0,771 | 1,65 | 0,975 | 0,0425 | 0,65 |
| PC.ae.C34.0 | 0,99 | 0,067 | 0,75 | 0,286 | 0,0455 | 0,76 |
| PC.ae.C36.1 | 4,86 | 0,384 | 3,95 | 1,19 | 0,0481 | 0,81 |
| PC.aa.C34.2 | 237,96 | 71,248 | 191,67 | 47,025 | 0,0496 | 0,81 |

**A**

**B**

| **Metabolite** | **Mean Conc. Control [µM]** | **SD Controls** | **Mean Conc. NDMM [µM]** | **SD NDMM** | **p-value: NDMM-Control** | **Fold Change: NDMM-Control** |
| --- | --- | --- | --- | --- | --- | --- |
| Sarcosine | 5,32 | 2,845 | 1,68 | 0,788 | 2,71E-13 | 0,32 |
| Trp | 74,03 | 15,756 | 47,55 | 9,145 | 8,82E-10 | 0,64 |
| PC.aa.C36.5 | 28,90 | 7,891 | 10,93 | 5,315 | 1,35E-08 | 0,38 |
| Asp | 2,48 | 2,013 | 14,35 | 15,843 | 2,92E-08 | 5,79 |
| PC.aa.C38.3 | 37,94 | 4,674 | 23,47 | 9,781 | 2,23E-07 | 0,62 |
| lysoPC.a.C20.3 | 1,76 | 0,367 | 0,99 | 0,408 | 3,67E-07 | 0,56 |
| ADMA | 0,36 | 0,121 | 0,60 | 0,166 | 4,38E-07 | 1,67 |
| lysoPC.a.C18.0 | 24,75 | 3,017 | 15,06 | 5,814 | 1,55E-06 | 0,61 |
| lysoPC.a.C16.0 | 99,87 | 5,623 | 66,89 | 21,594 | 1,91E-06 | 0,67 |
| C2 | 5,20 | 1,722 | 9,60 | 3,962 | 3,23E-06 | 1,85 |
| SDMA | 0,53 | 0,291 | 1,21 | 0,871 | 3,40E-06 | 2,28 |
| PC.aa.C36.3 | 91,36 | 9,282 | 64,99 | 23,564 | 7,21E-06 | 0,71 |
| PC.aa.C36.6 | 0,90 | 0,317 | 0,45 | 0,217 | 1,32E-05 | 0,50 |
| lysoPC.a.C26.1 | 0,12 | 0,016 | 0,07 | 0,05 | 2,19E-05 | 0,58 |
| C18 | 0,03 | 0,007 | 0,06 | 0,038 | 2,68E-05 | 2,00 |
| C0 | 32,62 | 4,063 | 47,65 | 15,727 | 2,87E-05 | 1,46 |
| PC.aa.C34.4 | 1,35 | 0,4 | 0,77 | 0,46 | 0,0001 | 0,57 |
| PC.aa.C38.5 | 43,90 | 5,757 | 30,18 | 9,507 | 0,0001 | 0,69 |
| C18.1 | 0,09 | 0,003 | 0,17 | 0,078 | 0,0001 | 1,89 |
| PC.aa.C40.6 | 22,57 | 4,75 | 14,94 | 5,164 | 0,0002 | 0,66 |
| alpha.AAA | 1,68 | 0,89 | 0,99 | 1,045 | 0,0004 | 0,59 |
| lysoPC.a.C16.1 | 3,03 | 0,972 | 2,13 | 1,028 | 0,0006 | 0,70 |
| Leu | 202,25 | 34,655 | 142,23 | 43,521 | 0,0006 | 0,70 |
| PC.aa.C38.6 | 60,36 | 11,93 | 42,19 | 13,111 | 0,0007 | 0,70 |
| Serotonin | 0,27 | 0,141 | 0,16 | 0,307 | 0,0008 | 0,59 |
| Creatinine | 73,72 | 43,501 | 161,29 | 159,896 | 0,0010 | 2,19 |
| PC.aa.C36.2 | 147,17 | 49,539 | 112,05 | 34,377 | 0,0015 | 0,76 |
| lysoPC.a.C18.2 | 22,31 | 5,522 | 14,80 | 6,327 | 0,0019 | 0,66 |
| Tyr | 103,99 | 32,14 | 79,80 | 20,072 | 0,0021 | 0,77 |
| PC.aa.C36.1 | 37,18 | 5,3 | 29,19 | 14,501 | 0,0023 | 0,79 |
| PC.aa.C24.0 | 0,06 | 0,033 | 0,11 | 0,059 | 0,0026 | 1,83 |
| PC.aa.C40.5 | 8,24 | 1,336 | 6,22 | 2,288 | 0,0027 | 0,75 |
| PC.aa.C42.0 | 0,37 | 0,14 | 0,28 | 0,099 | 0,0027 | 0,76 |
| Val | 339,60 | 40,583 | 263,75 | 60,996 | 0,0030 | 0,78 |
| PC.aa.C34.3 | 12,03 | 1,438 | 8,85 | 4,418 | 0,0037 | 0,74 |
| Ser | 111,68 | 48,944 | 146,51 | 49,318 | 0,0052 | 1,31 |
| PC.ae.C44.5 | 1,16 | 0,354 | 0,87 | 0,366 | 0,0058 | 0,75 |
| PC.ae.C38.0 | 1,77 | 0,083 | 1,23 | 0,429 | 0,0067 | 0,69 |
| C18.2 | 0,03 | 0,007 | 0,05 | 0,027 | 0,0090 | 1,67 |
| PC.ae.C44.4 | 0,27 | 0,057 | 0,21 | 0,084 | 0,0107 | 0,78 |
| PC.ae.C44.6 | 0,75 | 0,249 | 0,59 | 0,227 | 0,0120 | 0,79 |
| Thr | 122,86 | 60,501 | 101,94 | 32,732 | 0,0167 | 0,83 |
| PC.aa.C42.6 | 0,36 | 0,061 | 0,29 | 0,084 | 0,0170 | 0,81 |
| lysoPC.a.C18.1 | 16,21 | 2,237 | 13,12 | 4,959 | 0,0172 | 0,81 |
| C14.1 | 0,07 | 0,007 | 0,10 | 0,042 | 0,0184 | 1,43 |
| C16 | 0,10 | 0,008 | 0,14 | 0,061 | 0,0197 | 1,40 |
| PC.aa.C32.2 | 2,53 | 0,771 | 1,87 | 1,157 | 0,0200 | 0,74 |
| PC.aa.C32.3 | 0,34 | 0,083 | 0,27 | 0,107 | 0,0215 | 0,79 |
| lysoPC.a.C20.4 | 4,96 | 0,379 | 3,94 | 2,011 | 0,0259 | 0,79 |
| Ile | 113,68 | 17,898 | 89,62 | 23,798 | 0,0264 | 0,79 |
| PC.ae.C38.1 | 0,38 | 0,157 | 0,68 | 0,448 | 0,0280 | 1,79 |
| PC.aa.C34.1 | 146,42 | 13,868 | 130,07 | 37,019 | 0,0372 | 0,89 |
| PC.ae.C40.1 | 0,93 | 0,15 | 0,76 | 0,278 | 0,0391 | 0,82 |
| Gly | 295,08 | 126,917 | 387,44 | 174,11 | 0,0418 | 1,31 |
| Glu | 142,80 | 42,147 | 201,47 | 93,121 | 0,0419 | 1,41 |
| Kynurenine | 2,24 | 0,964 | 3,20 | 1,626 | 0,0444 | 1,43 |
| PC.aa.C34.2 | 237,96 | 71,248 | 204,66 | 49,631 | 0,0496 | 0,86 |

**C**

| **Metabolite** | **Mean Conc. Control [µM]** | **SD Controls** | **Mean Conc. RRMM [µM]** | **SD RRMM** | **p-value: RRMM-Control** | **Fold Change: RRMM-Control** |
| --- | --- | --- | --- | --- | --- | --- |
| Sarcosine | 5,32 | 2,845 | 1,72 | 0,921 | 3,10E-08 | 0,32 |
| C18 | 0,03 | 0,007 | 0,06 | 0,022 | 5,58E-07 | 2,00 |
| Asp | 2,48 | 2,013 | 11,39 | 6,422 | 6,51E-06 | 4,59 |
| alpha.AAA | 1,68 | 0,89 | 0,64 | 0,384 | 1,88E-05 | 0,38 |
| lysoPC.a.C26.1 | 0,12 | 0,016 | 0,06 | 0,038 | 2,19E-05 | 0,50 |
| ADMA | 0,36 | 0,121 | 0,71 | 0,48 | 0,0001 | 1,97 |
| Serotonin | 0,27 | 0,141 | 0,09 | 0,102 | 0,0003 | 0,33 |
| C18.1 | 0,09 | 0,003 | 0,15 | 0,048 | 0,0007 | 1,67 |
| Leu | 202,25 | 34,655 | 137,99 | 39,149 | 0,0008 | 0,68 |
| Trp | 74,03 | 15,756 | 55,04 | 17,723 | 0,0020 | 0,74 |
| Tyr | 103,99 | 32,14 | 78,55 | 17,395 | 0,0021 | 0,76 |
| PC.aa.C36.5 | 28,90 | 7,891 | 16,78 | 6,58 | 0,0022 | 0,58 |
| PC.aa.C38.3 | 37,94 | 4,674 | 29,21 | 7,183 | 0,0026 | 0,77 |
| PC.aa.C42.0 | 0,37 | 0,14 | 0,26 | 0,09 | 0,0027 | 0,70 |
| PC.aa.C36.3 | 91,36 | 9,282 | 74,26 | 15,501 | 0,0033 | 0,81 |
| PC.aa.C36.1 | 37,18 | 5,3 | 30,07 | 5,636 | 0,0039 | 0,81 |
| Val | 339,60 | 40,583 | 260,53 | 65,176 | 0,0043 | 0,77 |
| PC.ae.C44.5 | 1,16 | 0,354 | 0,82 | 0,32 | 0,0058 | 0,71 |
| PC.ae.C44.6 | 0,75 | 0,249 | 0,54 | 0,176 | 0,0069 | 0,72 |
| Ile | 113,68 | 17,898 | 82,69 | 20,785 | 0,0099 | 0,73 |
| PC.ae.C44.4 | 0,27 | 0,057 | 0,21 | 0,06 | 0,0117 | 0,78 |
| PC.aa.C36.2 | 147,17 | 49,539 | 120,60 | 23,379 | 0,0162 | 0,82 |
| Thr | 122,86 | 60,501 | 95,58 | 29,248 | 0,0167 | 0,78 |
| PC.aa.C34.3 | 12,03 | 1,438 | 9,36 | 2,713 | 0,0200 | 0,78 |
| Ser | 111,68 | 48,944 | 138,26 | 34,809 | 0,0268 | 1,24 |
| C16 | 0,10 | 0,008 | 0,13 | 0,046 | 0,0272 | 1,30 |
| Cit | 39,76 | 17,882 | 28,37 | 8,455 | 0,0277 | 0,71 |
| PC.aa.C36.6 | 0,90 | 0,317 | 0,62 | 0,21 | 0,0290 | 0,69 |
| PC.aa.C40.6 | 22,57 | 4,75 | 17,42 | 4,608 | 0,0305 | 0,77 |
| SDMA | 0,53 | 0,291 | 0,92 | 0,783 | 0,0360 | 1,74 |
| PC.aa.C34.1 | 146,42 | 13,868 | 126,82 | 26,337 | 0,0372 | 0,87 |
| Gly | 295,08 | 126,917 | 396,05 | 150,194 | 0,0418 | 1,34 |

**D**

| **Metabolite** | **Mean Conc. NDMM [µM]** | **SD NDMM** | **Mean Conc. MGUS [µM]** | **SD MGUS** | **p-value: RRMM-MGUS** | **Fold Change: RRMM-MGUS** |
| --- | --- | --- | --- | --- | --- | --- |
| C0 | 34,90 | 7,04 | 47,65 | 15,727 | 0,0032 | 1,37 |
| C2 | 6,69 | 1,451 | 9,60 | 3,962 | 0,0094 | 1,43 |
| PC.ae.C36.1 | 3,95 | 1,19 | 5,62 | 2,344 | 0,0167 | 1,42 |
| ADMA | 0,47 | 0,161 | 0,60 | 0,166 | 0,0234 | 1,28 |
| PC.aa.C34.1 | 106,06 | 25,772 | 130,07 | 37,019 | 0,0372 | 1,23 |
| Glu | 138,00 | 106,502 | 201,47 | 93,121 | 0,0439 | 1,46 |
| PC.ae.C34.0 | 0,75 | 0,286 | 1,01 | 0,413 | 0,0455 | 1,35 |
| PC.ae.C44.5 | 1,06 | 0,307 | 0,87 | 0,366 | 0,0473 | 0,82 |

**E**

| **Metabolite** | **Mean Conc. RRMM [µM]** | **SD RRMM** | **Mean Conc. MGUS [µM]** | **SD MGUS** | **p-value: RRMM-MGUS** | **Fold Change: RRMM-MGUS** |
| --- | --- | --- | --- | --- | --- | --- |
| PC.aa.C36.1 | 30,07 | 5,636 | 23,00 | 6,751 | 0,0070 | 1,31 |
| PC.ae.C34.0 | 1,06 | 0,248 | 0,75 | 0,286 | 0,0088 | 1,41 |
| PC.ae.C36.1 | 5,33 | 1,468 | 3,95 | 1,19 | 0,0167 | 1,35 |
| ADMA | 0,71 | 0,48 | 0,47 | 0,161 | 0,0234 | 1,51 |
| PC.ae.C44.6 | 0,54 | 0,176 | 0,77 | 0,303 | 0,0252 | 0,70 |
| C18 | 0,06 | 0,022 | 0,04 | 0,016 | 0,0293 | 1,50 |
| PC.aa.C34.1 | 126,82 | 26,337 | 106,06 | 25,772 | 0,0372 | 1,20 |
| PC.ae.C44.5 | 0,82 | 0,32 | 1,06 | 0,307 | 0,0436 | 0,77 |

**F**

| **Metabolite** | **Mean Conc. RRMM [µM]** | **SD RRMM** | **Mean Conc. NDMM [µM]** | **SD NDMM** | **p-value: RRMM-NDMM** | **Fold Change: RRMM-NDMM** |
| --- | --- | --- | --- | --- | --- | --- |
| lysoPC.a.C20.3 | 1,57 | 0,63 | 0,99 | 0,408 | 0,0005 | 1,59 |
| PC.aa.C36.5 | 16,78 | 6,58 | 10,93 | 5,315 | 0,0022 | 1,54 |
| lysoPC.a.C18.0 | 21,63 | 7,417 | 15,06 | 5,814 | 0,0027 | 1,44 |
| lysoPC.a.C17.0 | 1,78 | 0,521 | 1,32 | 0,547 | 0,0087 | 1,35 |
| PC.aa.C38.3 | 29,21 | 7,183 | 23,47 | 9,781 | 0,0121 | 1,24 |
| PC.aa.C36.6 | 0,62 | 0,21 | 0,45 | 0,217 | 0,0133 | 1,38 |
| lysoPC.a.C16.0 | 86,82 | 25,407 | 66,89 | 21,594 | 0,0148 | 1,30 |
| Creatinine | 85,96 | 46,585 | 161,29 | 159,896 | 0,0171 | 0,53 |
| C2 | 7,03 | 5,356 | 9,60 | 3,962 | 0,0208 | 0,73 |
| C0 | 36,83 | 15,119 | 47,65 | 15,727 | 0,0239 | 0,77 |
| PC.aa.C38.5 | 36,65 | 8,809 | 30,18 | 9,507 | 0,0252 | 1,21 |
| lysoPC.a.C20.4 | 5,02 | 1,518 | 3,94 | 2,011 | 0,0256 | 1,27 |
| PC.aa.C34.4 | 1,04 | 0,414 | 0,77 | 0,46 | 0,0325 | 1,35 |
