# Supplementary figures and images for: The metabolomic plasma profile of myeloma patients is considerably different from healthy subjects and reveals potential new therapeutic targets
Source: PLoS One. 2018 Aug 10;13(8):e0202045. doi: 10.1371/journal.pone.0202045 (PMC6086450; doi:10.1371/journal.pone.0202045)

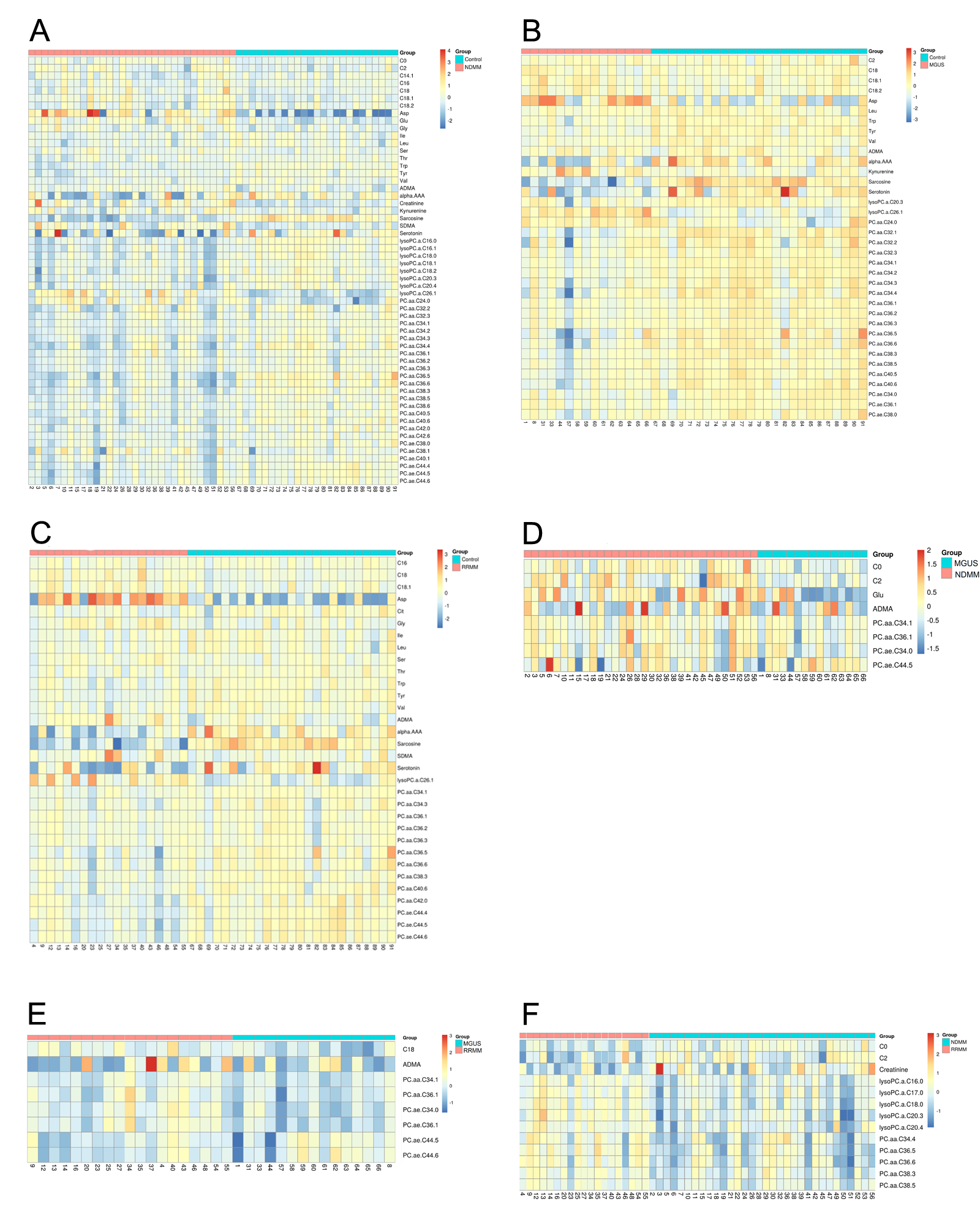

Supplement: S1 Fig — A) heat maps of metabolites between healthy controls and NDMM. B) heat maps of metabolites between healthy controls and MGUS. C) heat maps of metabolites between healthy controls and RRMM. D) heat maps of metabolites between MGUS and NDMM. E) heat maps of metabolites between MGUS and RRMM. F) heat maps of metabolites between NDMM and RRMM. (TIF) [file pone.0202045.s005.tif]
